# Investigating socioeconomic deprivation and antibiotic prescribing among older medicare patients using an instrumental variable approach

**DOI:** 10.1017/ash.2025.185

**Published:** 2025-05-13

**Authors:** Mayar Al Mohajer, David Slusky, David Nix, Catia Nicodemo

**Affiliations:** 1 Department of Medicine, Baylor College of Medicine, Houston, TX, USA; 2 Department of Continuous Education, University of Oxford, Oxford, UK; 3 Department of Primary Health Care, University of Oxford, Oxford, UK; 4 Department of Economics, University of Kansas, Lawrence, KS, USA; 5 National Bureau of Economic Research, Cambridge, MA, USA; 6 IZA - Institute of Labor Economics, Bonn, Germany; 7 Department of Pharmacy Practice and Science, University of Arizona, Tucson, AZ, USA; 8 Department of Economics, University of Verona, Verona, Italy

## Abstract

**Background:**

Socioeconomic deprivation has been associated with antibiotic overprescription in the US; however, prior studies could not quantify a causal relationship due to endogeneity. This study examines how socioeconomic deprivation is related to the rate of antibiotic days supplied to older Medicare Part D beneficiaries, utilizing an Instrumental Variable (IV) approach.

**Methods:**

Data from the Medicare Part D and the Social Deprivation Index (SDI) repositories were analyzed. To address potential endogeneity and omitted variable bias in the relationship between SDI and antibiotic prescribing, we used the maximum Earned Income Tax Credit as an IV. Bivariate Moran’s *I* assessed the spatial correlation between SDI and antibiotic prescribing across geographic regions. The IV analysis then examined the relationship between predicted SDI and antibiotic days supplied (ln). Linear regression models estimated associations between SDI and its components, and antibiotic days supplied, adjusting for prescriber, beneficiary, and geographic factors.

**Results:**

Among 161,164, there was no significant spatial dependence between SDI and antibiotic days supplied (*P* = 0.0656). In the IV model, a one-unit increase in SDI was associated with a 0.582 (SE = 0.164, *P* < 0.0005) increase in antibiotic days supplied (ln). Higher unemployment and single-parent family rates were linked to increased antibiotic days supplied, while crowded housing was associated with a reduction.

**Conclusion:**

This study identified that socioeconomic deprivation may influence antibiotic days supplied to Medicare Part D beneficiaries. Findings highlight the need for targeted public health interventions to address the socioeconomic factors contributing to excess antibiotic use.

## Introduction

Antibiotic overuse is a known risk factor for antimicrobial resistance and *Clostridioides difficile* (*C. difficile*) infections. According to the Centers for Disease Control and Prevention, the cumulative number of infections due to multidrug-resistant organisms *and C. difficile* is estimated to be 3 million cases yearly, resulting in 48,000 deaths and over $4.6 billion in excess costs.^1^ By 2050, the total number of deaths secondary to multidrug-resistant organisms is projected to exceed 10 million annually.^2^

Older individuals ( ≥65 years) are disproportionately prescribed a high rate of antimicrobials in the outpatient setting and are at an elevated risk of developing antibiotic-related side effects.^3^ Among beneficiaries of the Medicare Part D Program, who represent 70% of all Medicare, there are significant variations in prescribing patterns of antibiotics across different geographic regions.^4^ Enrollees in the US South are prescribed a higher rate of antibiotics per 100 beneficiaries (median 47.1 vs. 43.2 in the Northeast). The etiology of this variation has been hypothesized in one study from Texas to be connected to socioeconomic factors leading to underuse when antibiotics are required and overuse where they may be unnecessary.^5^

Several studies have evaluated the association between socioeconomic factors and clinical outcomes, focusing on deprivation indices. One principal metric is the social deprivation index (SDI), a composite measure of several socioeconomic factors, such as education, income, and housing.^6^ Higher SDI indicates higher deprivation, which is related to worse clinical results, including lower life expectancy in patients with cardiovascular diseases.^7^

Among patients with infectious diseases, the relationship between SDI and antibiotic prescription was found to be complex. Data from Massachusetts indicated fewer visits for respiratory tract infections among patients younger than 65 with high SDI.^8^ In Texas,^5^ more antibiotic claims were associated with higher SDI scores in rural areas, while lower scores were observed in urban areas.

The previous literature has focused on the association between socioeconomic deprivation and antibiotic utilization but was unable to establish a causal relationship, given the design of these studies and the unmeasured confounders, such as access, education, comorbidities, and provider and patient behaviors. Consequently, it is crucial to disentangle the multifaceted relationship between SDI and antibiotic prescribing. The instrument variable (IV) approach has been utilized previously to isolate the effect of socioeconomic factors on health outcomes and provide more accurate estimates of this link when randomization is not possible.^9^

Some studies have employed the state-level maximum Earned Income Tax Credit (EITC), a refundable tax credit for low- to moderate-income working individuals and families, as an IV to predict socioeconomic factors and isolate their impact on health outcomes.^9,10^ While the EITC for each family is affected by individual income and family structure, the maximum EITC in each state is determined solely by state and federal policies.^11^ It was chosen over alternative instruments, such as wage rates or Medicaid expansion, due to its strong correlation with socioeconomic conditions and minimal direct association with specific health behaviors.

The study aims to explore the relationship between SDI and antibiotic days supplied per 100 beneficiaries using the maximum EITC as the SDI instrument. This approach overcomes the limitations of previous observational studies and analyzes how the SDI and its components influence antibiotic prescription among older patients in the US.

## Study data and methods

### Data

This study utilized several repositories: 1) The Medicare Part D Prescribers datasets (FY 2013–2022), including provider-level and drug-specific data, 2) The American Community Survey (ACS), 3) A dataset of teaching hospitals, 4) The Doctor and Clinicians repository, and 5) The US Best Medical Schools for Research dataset.

Medicare Part D Prescribers by Provider datasets included provider characteristics (e.g., National Provider Identifier [NPI], gender), practices (e.g., address, metropolitan area, number of beneficiaries), and patients (e.g., average age, gender distribution, race, ethnicity, average risk score).^12^ The Medicare Part D Prescribers by Provider and Drug datasets contained information on antibiotics prescribed, including days supplied, total claims, and cost.^13^

The ACS dataset provided the SDI score at the census tract level on a scale from 0 to 100, with higher scores indicating greater deprivation.^14^ Additionally, it included the percentages and normalized values for the seven SDI components: poverty, low education, single-parent households, unemployment, renter-occupied housing units, crowding, and no vehicle. Since the SDI was not available annually, SDI values for each year were assigned based on the nearest available year; for example, SDI values for 2020 and later years were assigned using 2019 SDI data.

The remaining three datasets were used to determine whether the practice was in a ZIP code with a teaching hospital (academic location),^15^ assess provider medical experience (since graduation),^16^ and evaluate their graduate school rank.^17^

These datasets were merged based on the provider NPI and the practice geographic location (Zip code or census tract). Centers for Medicare & Medicaid Services (CMS) provided zip code details, while census tract information was extracted from addresses using the *tidygeocoder* package.^18^ Additional details on the database structure, variable definitions, and methods used for merging these datasets can be found in our previous work.^5,19^

### Inclusion and exclusion criteria

The study included providers from the following specialties: Family Medicine, Internal Medicine, Hospitalist, Emergency Medicine, mid-level providers, and students. Providers from overseas or US territories were excluded, as were those with missing data for key variables. Claims for antivirals, antifungals, antiparasitics, and topical antibiotics were also excluded, as the study focused specifically on antibiotics most commonly associated with bacterial resistance. While resistance to these agents is essential, excluding them allowed for a targeted analysis of bacterial resistance.

### Study outcomes

The primary outcome (Y) was the rate of antibiotic days supplied per 100 beneficiaries. Secondary outcomes comprised the rate of antibiotic claims per 100 days, the number of days per claim, and the cost per 100 beneficiaries. The rate of days supplied, claims, and cost were log-transformed using the natural logarithm (ln) for analysis, as these variables were not normally distributed.

### Social deprivation index and IV

This study examines how socioeconomic deprivation relates to antibiotic prescribing using the composite normalized SDI score and its seven components (see above) as a proxy for disadvantage.^14^ Given the challenge of unmeasured factors that could affect both SDI and prescribing—such as healthcare access, income, and comorbidities—we applied an IV approach to strengthen causal inference.

The EITC is a state-level policy shaping economic conditions that influence SDI. However, a valid IV must vary independently of prescribing behaviors. Thus, maximum EITC per state was selected as the IV, capturing long-term economic conditions.

**Figure 1.**
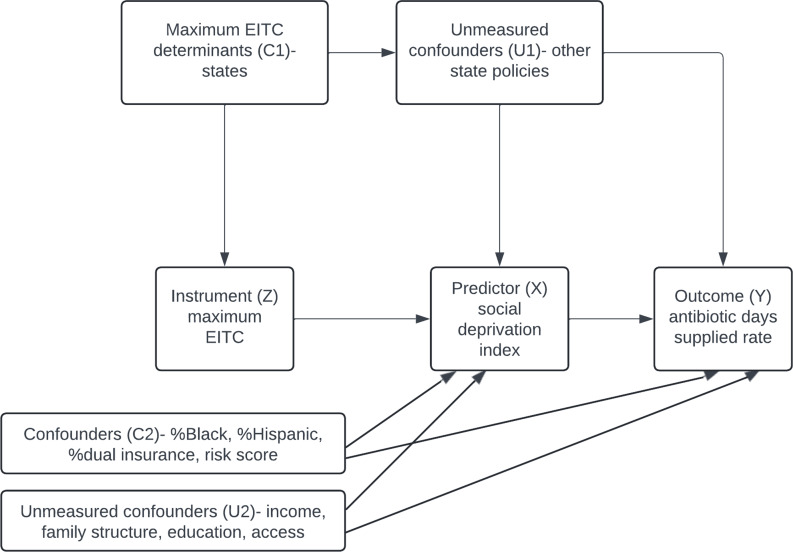
This directed acyclic graph illustrates how the maximum Earned Income Tax Credit (EITC) is used as an instrument (Z) to estimate the effect of the Social Deprivation Index (SDI) (X) on the rate of antibiotic days supplied (Y). The instrumental variable (IV) approach is based on three key assumptions: 1) Relevance: EITC is strongly correlated with SDI. 2) Exclusion Restriction: EITC influences the rate of antibiotic days supplied only through SDI, with no direct effect. 3) No Unmeasured Confounders: Confounders between EITC and the outcome are addressed, including state policies and other variables in the model. EITC: Earned Income Tax Credit; SDI: Social Deprivation Index.

A standard regression model could be biased because of factors like healthcare access, which is associated with both SDI and prescribing (Figure 1). The IV approach improves validity by first estimating SDI using maximum EITC and then assessing its relationship with prescribing. For this method to be valid, maximum EITC must be strongly associated with SDI while affecting prescribing only through SDI, assumptions supported by statistical tests. By using IV, this study provides a more robust estimate of how socioeconomic deprivation may influence antibiotic prescribing, offering insights beyond standard regression models.^20^

### Covariates

We adjusted for the provider’s state (C1) (Figure 1), to account for state-level policies affecting SDI and antibiotic use. Additional confounders (C2) included claim year, provider demographics, specialty, academic location, metropolitan status, and patient characteristics (age, risk score, sex, race, and dual insurance status). Geographic regions were based on US Census Bureau divisions, with urban/rural areas classified by Rural_Urban Comuting Area (RUCA).^21^

### Statistical analysis

To prevent underestimation of antibiotic prescriptions, CMS-suppressed values for claims under 11 were imputed as five. Complete case analysis was used for other missing data to avoid introducing further bias.

We fitted fixed-effects multiple Ordinary Least Squares (OLS) and two-stage Instrumental Variable (IV) regression models to assess the relationship between SDI (or its components) and the study outcomes. These models accounted for the aforementioned confounders (C1 and C2). The IV model followed a two-stage process: first, SDI was regressed on the instrument (maximum EITC) to obtain predicted SDI values; second, these predicted values were used to estimate the association between SDI and antibiotic days supplied.

The analysis was conducted using the *ivreg* package in R.^22^ The weak instrument (*F*) test and the Wu-Hausman test were used to evaluate the instrument’s strength and the presence of endogeneity, respectively. Given the large sample size, an alpha cut-off of 0.0005 was applied, calculated using the Good Standard value formula (0.05/√(n/100)).^23^

To determine whether the observed relationship is influenced by geographic factors, spatial autocorrelations within and between the predicted SDI (from the IV model) and the antibiotic days supplied rate per 100 beneficiaries were evaluated using Local Indicators of Spatial Association (LISA) cluster mapping and global Moran’s *I* analyses. The analysis was performed using R version 4.2.2 (Vienna, Austria).

## Results

### Study characteristics

This study included 161,164 providers across 25,007 census tracts. Characteristics of providers, patients, and antibiotic claims are presented in Table 1. The median age of beneficiaries was 70.2 years, and most were female (60.8%). Blacks and Hispanics represented 26.2% and 10.5% of all patients, respectively.

**Table 1.** Characteristics of providers, patients, and antibiotic claims (FY 2013–2022)

Characteristics	
Provider (n = 161,164)	
**Gender**	n (%)
Male	72,377 (44.9)
Female	88,787 (55.1)
**Specialty**	n (%)
Emergency Medicine	15,733 (9.8)
Family Medicine	32,181 (20.0)
Hospitalist	4,523 (2.8)
Internal Medicine	35,507 (22.0)
Nurse Practitioner	40,198 (24.9)
Physician Assistant	23,882 (14.8)
Student	9,140 (5.7)
**Academic location**	n (%)
Yes	61,570 (38.2)
No	99,594 (61.8)
	Mean (SD)
Years of professional experience	11.0 (9.9)
**Geography**	
*Metropolitan area*	n (%)
Yes	138,914 (86.2)
No	22,250 (13.8)
**Region**	n (%)
Northeast	35,955 (22.3)
Midwest	39,384 (24.4)
South	53,952 (33.5)
West	31,873 (19.8)
**Patients**	Mean (SD)
Beneficiaries per provider	217 (217)
Age (years)	70.2 (3.9)
% Female beneficiaries per provider	60.8 (8.9)
% Black beneficiaries per provider	16.2 (18.1)
% Hispanic beneficiaries per provider	10.5 (16.0)
% With dual insurance	36.1 (18.6)
Risk score	1.6 (0.6)
**SDI**	n (%)
Providers in quintile 1 -SDI [89,100] (most deprived)	30,659 (19.0)
Providers in quintile 2 -SDI [73, 89)	32,533 (20.2)
Providers in quintile 3 -SDI [54, 73)	33,356 (20.7)
Providers in quintile 4 -SDI [32, 54)	31,735 (19.7)
Providers in quintile 5 -SDI [1, 32) (least deprived)	32,881 (20.4)
**Claims**	Mean (SD)
Total antibiotic days supplied per provider	638 (1,185)
Rate of antibiotic days supplied/ 100 beneficiaries per provider	278 (418)
Total antibiotic claims per provider	69 (120)
Rate of claims/ 100 beneficiaries per provider	28.5 (25.6)
Antibiotic days per claim	9.5 (7.1)
Antibiotic cost per provider	1,361 (4.959)
Rate of antibiotic cost/ 100 beneficiaries per provider	680 (6,866)

SDI: Social Deprivation Index; SD: Standard Deviation.

### Predicted SDI

The first stage of the IV analysis indicated that the ln maximum EITC was predictive of SDI (estimate –0.0652, *P* < 0.0005). The *F* statistic for the first stage was well above the standard cut-off of 10, indicating that the maximum EITC was a strong instrument for SDI.

**Figure 2.**
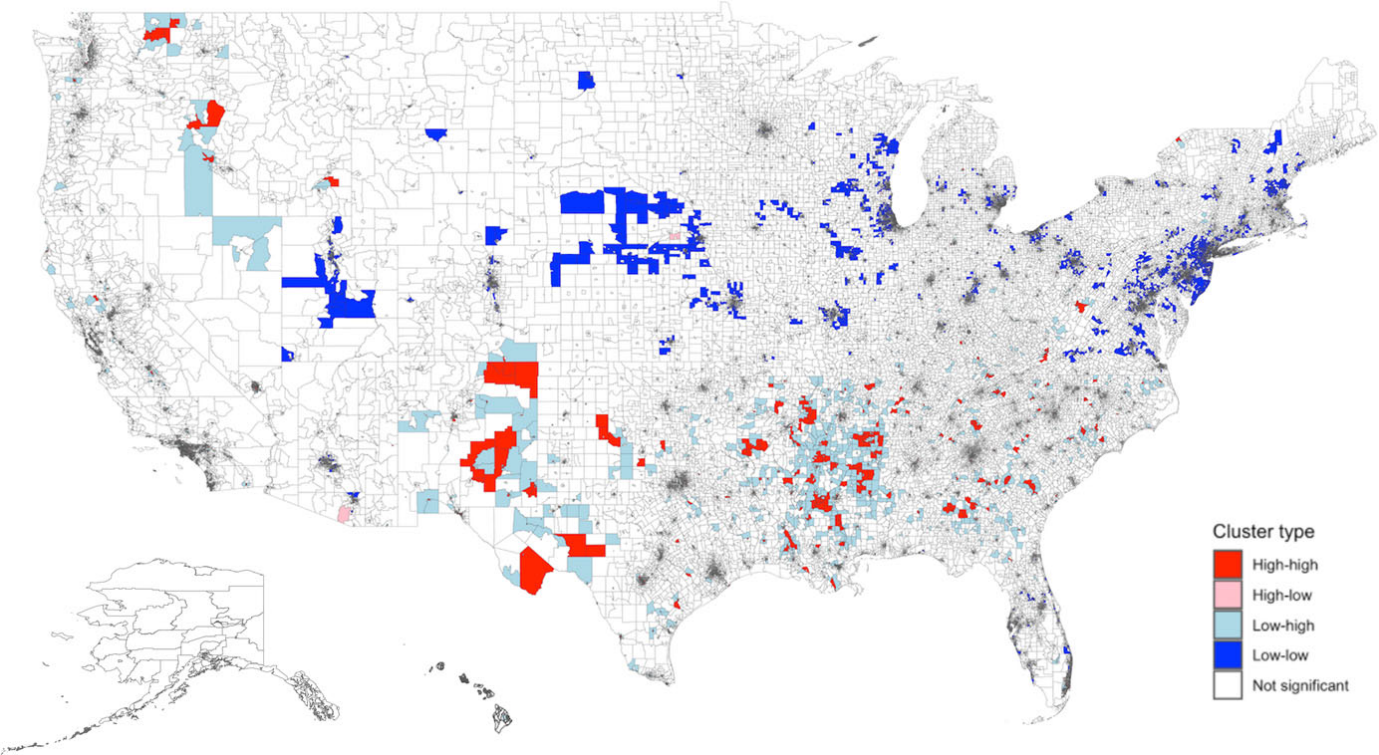
Spatial distribution and empirical Bayes FIPS cluster map of Predicted SDI in the US. There was significant spatial clustering of predicted SDI in the US (Moran’s *I* global statistic 0.55, *P* < 0.0001). The distribution included 2,100 high-high, 204 high-low, 3,463 low-high, 5,264 low-low, and 61,782 non-significant clusters. “High-high” clusters represent areas with high SDI values, and “low-low” clusters represent areas with low SDI values. High-high clusters were largely located in the South. Low-low clusters were mainly seen in the Northeast and Midwest regions. Predicted SDI values were obtained from the instrument value model using maximum EITC. The adjusted values were then obtained after regressing on other variables. SDI: Social Deprivation Index; FIPS: Federal Information Processing Series; LISA: Local Indicators of Spatial Association.

Predicted SDI (values obtained from the IV model) had significant spatial clustering across the US (Moran’s *I* global statistic 0.55, *P* < 0.0001). This indicates that census tracts with similar SDI values tend to cluster geographically, reflecting underlying socioeconomic patterns. A total of 2,100 census tracts with high SDI were adjacent to other neighborhoods with high SDI (Figure 2). These high-high clusters were primarily located in the South. Conversely, there were 5,264 low-low clusters, mostly in the Northeast and Midwest regions.

### Primary Outcome

The mean antibiotic days supplied per provider for 100 beneficiaries was 278 (SD 418) (Table 1). The rate of days supplied was highest in the South (mean 297), in non-metropolitan areas (373), and among internal medicine providers (314). Penicillins and fluoroquinolones had the highest mean days supplied per provider (185 and 173 days, respectively). The rate of days supplied decreased from 356 in FY2013 to 322 in FY2022.

**Figure 3.**
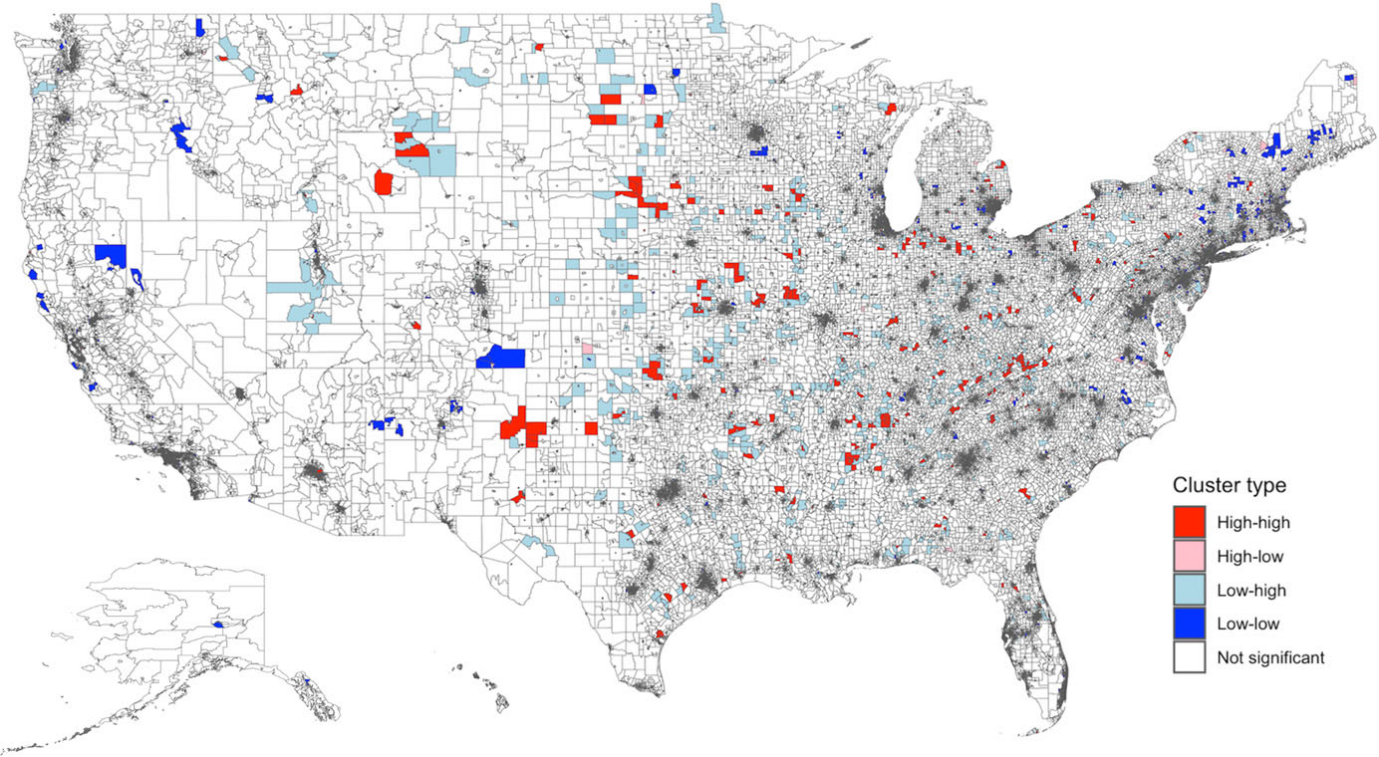
Spatial distribution and empirical Bayes FIPS cluster map of antibiotic days supplied rate (ln) in the US. There was significant spatial clustering of antibiotic rate supplied in the US (Moran’s *I* global statistic 0.08, *P* < 0.0001). The distribution included 794 high-high, 559 high-low, 1,914 low-high, 2,395 low-low, and 67,151 non-significant clusters. “High-high” clusters represent areas with a high rate of antibiotic days supplied, and “low-low” clusters reflect areas with a low rate of antibiotic days supplied. High-high clusters were largely located in the South and Midwest. Low-low clusters were mainly seen in the Northeast and California. FIPS: Federal Information Processing Series; LISA: Local Indicators of Spatial Association.

There was significant spatial clustering of the rate of antibiotic days supplied (ln) (Moran’s *I* global statistic 0.08, *P* < 0.0001). Figure 3 shows the spatial distribution of antibiotic days supplied with 794 high-high and 2,395 low-low clusters. High-high clusters were largely located in the South and Midwest. Low-low clusters were mainly seen in the Northeast and California.

### Factors associated with primary outcome

**Figure 4.**
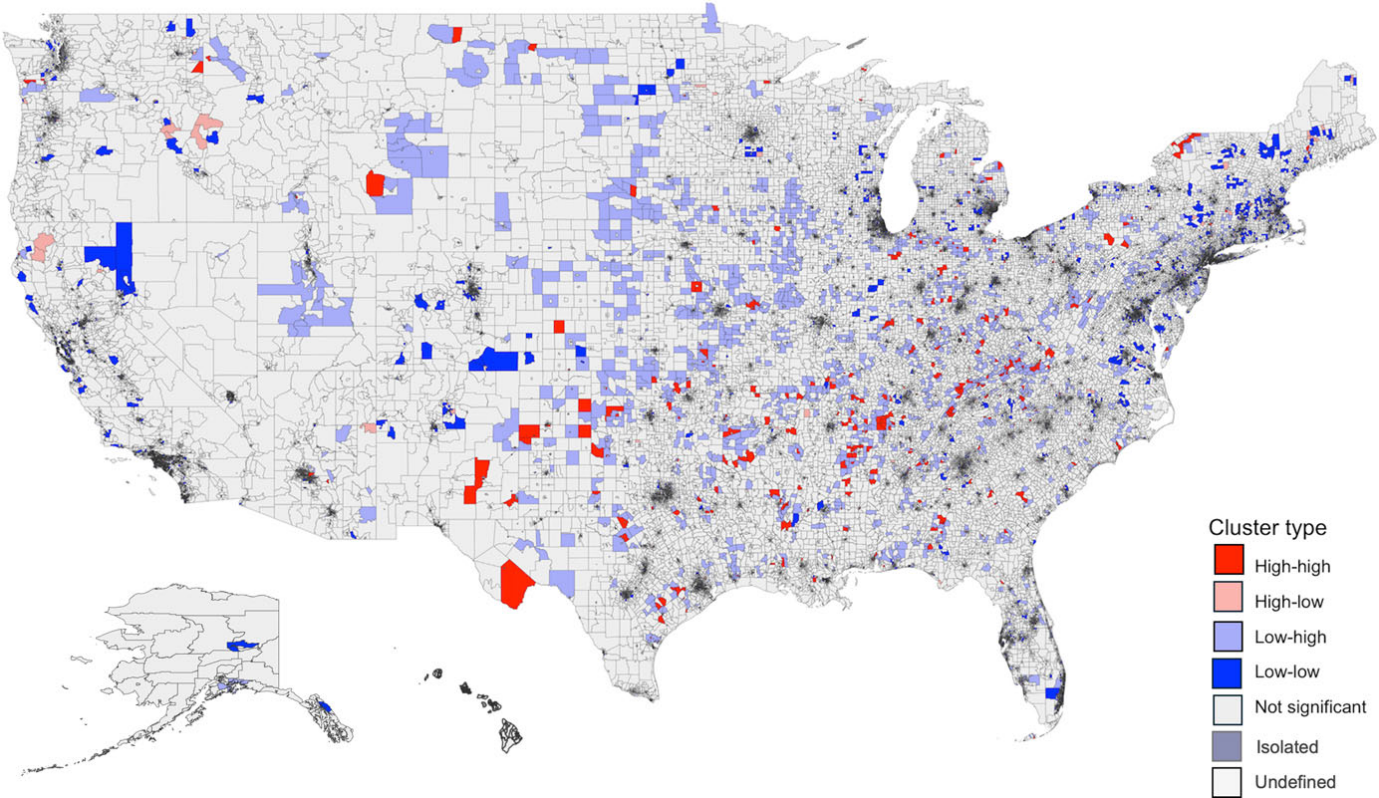
Bivariate LISA Map of predicted SDI and antibiotic days supplied rate (ln) Across US FIPS. Global Moran’s test showed no spatial dependence between predicted SDI and antibiotic days supplied rate (ln) in the US (*I* = 0.007, *P* = 0.0656). The distribution included 873 high-high, 4,292 low-low, 4,038 low-high, 947 high-low, 62,645 non-significant, 18 isolated, and 24 undefined clusters. “High-high” clusters in this map represent areas with both high SDI and high antibiotic days supplied, while “low-low” clusters represent areas with both low SDI and low antibiotic days supplied. High-high clusters were largely located in the South, and low-low clusters were mainly seen in the Northeast. LISA: Local Indicators of Spatial Association; SDI: Social Deprivation Index; FIPS: Federal Information Processing Series. Predicted SDI values were obtained from the instrument value model using maximum EITC. The adjusted values were then obtained after regressing on other variables.

There was no spatial dependence between predicted SDI (values from the IV model) and the ln rate of antibiotic days per 100 beneficiaries (global Moran’s test *I* = 0.007, *P* = 0.0656). The Bivariate LISA map (Figure 4) identified 873 high-high clusters (areas with both high SDI and high antibiotic use) primarily located in the South and 4,292 low-low clusters (areas with both low SDI and low antibiotic use) mostly in the Northeast.

Results from the unadjusted OLS model (Table 2) showed that SDI was associated with the antibiotic day supplied rate (ln) per 100 beneficiaries (estimate 0.00979, standard error [SE] 0.00115, *P* < 0.0005). Similarly, the IV model revealed that SDI predicted the ln antibiotic day supplied rate per 100 beneficiaries (estimate 0.582, SE 0.164, *P* < 0.0005). Higher day-supplied rates were seen in male providers, those with more experience, and those with higher beneficiary average age and risk scores. Conversely, it was lower among providers with a larger percentage of female, Black, and Hispanic patients.

**Table 2.** Association between the social deprivation index (SDI) and the rate of antibiotics days supplied per 100 beneficiaries (ln)

	SDI (first stage) Estimate (SE)	Ln Supplied Days Rate (OLS) (Unadjusted) Estimate (SE)#	Ln Supplied Days Rate (IV) Estimate (SE)#
SDI	–	0.00979* (0.00115)	0.582* (0.164)
Maximum EITC (ln)#	–0.0652* (0.00896)	–	–
Metropolitan	–0.319* (0.00295)	–0.113 (0.00322)	0.0699 (0.0525)
Prescriber Gender [Male]	–0.00260 (0.00227)	0.0208* (0.00246)	0.0223* (0.00293)
Provider experience	0.000631* (0.0000972)	0.0151* (0.000105)	0.0147* (0.000158)
Academic location	0.0994* (0.00705)	–0.139 (0.00764)	–0.196 (0.0183)
Metropolitan * Academic	0.235* (0.00733)	0.104 (0.00795)	–0.0303 (0.0395)
Beneficiary Average Age	–0.01673* (0.000307)	0.000343* (0.000333)	0.00991* (0.00278)
Beneficiary %Female	–0.00283* (0.000119)	–0.00715* (0.000129)	–0.00553* (0.000493)
Beneficiary %Black	0.00397* (0.0000681)	–0.00683* (0.0000740)	–0.00911 * (0.000658)
Beneficiary %Hispanic	0.00583* (0.0000738)	–0.00409* (0.0000803)	–0.00742* (0.000961)
Beneficiary %Dual Insurance	0.00828* (0.0000713)	0.00221* (0.0000779)	–0.00253 (0.00136)
Beneficiary Risk Score	0.00000522 (0.00202)	0.0730* (0.00219)	0.0730* (0.00331)

The model also controlled for the year, provider specialty, graduate school rank, and US state.

* indicates a *P* value < 0.0005 based on the Good standardized value (0.05/ 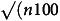 ).

# Log-transformed values (using the natural logarithm) were used to normalize the data.

SDI: Social Demographics Index; OLS: Ordinary Least squares; IV: Instrument variable; EITC: Earned Income Tax Credit.

The Weak instrument, *F* value 54.0 (*P* < 0.0001), suggests a strong instrument. The Wu-Hausman P<0.0001 indicates the presence of endogeneity, justifying the use of IV over the Ordinary Least squares models.

### Secondary outcomes

The average annual number of claims per 100 beneficiaries was 69, while the mean days per claim was 9.5. The average yearly cost of antibiotics per 100 beneficiaries was $680 (Table 1). The association between SDI and secondary outcomes is presented in Supplementary Tables 2–4. SDI predicted both the ln claim rate (estimate 0.517, SE 0.140, *P* < 0.0005, Supplementary Table 2) and the ln cost rate (estimate 1.19, SE 0.236, *P* < 0.0005, Supplementary Table 4); however, there was no association between SDI and days per claim (estimate 1.73, SE 0.978, *P* = 0.859, Supplementary Table 3). Notably, practices with a high proportion of Blacks and Hispanics had lower antibiotic claims and cost rates with no change in days per claim.

### SDI components

The ln maximum EITC predicted all seven SDI components (Table 3). The *F* statistics ranged from 14–441, indicating strong instruments. Practicing in census tracts with a high prevalence of single-parent families (estimate 0.00614, SE 0.00155, *P* < 0.0005) and unemployment (estimate 0.00620, SE 0.00156, *P* < 0.0005) led to a higher ln antibiotics days supplied rate. On the other hand, practicing in census tracts with a high prevalence of crowded housing predicted a lower rate (estimate –0.0139, SE 0.00378, *P* < 0.0005). Provider locations in census tracts with high percentages of poverty, less than 12 years of education, no vehicle, and renter-occupied units had similar antibiotic days supplied compared with those in other locations.

**Table 3.** Association between the individual components of the social deprivation index (SDI) and the rate of antibiotics days supplied (ln)^

	Weak instrument, F value$	Ln Supplied Days Rate (OLS) Estimate (SE)	Ln Supplied Days Rate (IV) Estimate (SE)
Population less than 100% federal poverty level	23.2	0.000423* (0.0000363)	0.0280 (0.00900)
Single-parent families with dependents <18 years	425	–0.0000864 (0.0000335)	0.00614* (0.00155)
Population ≥25 years <12 years of education	14.0	0.000456* (0.0000372)	–0.0336 (0.0124)
Households with no vehicle	25.6	–0.0000175 (0.0000384)	0.0273 (0.00871)
Households living in renter-occupied housing units	39.5	–0.000113 (0.0000387)	0.0234 (0.00693)
Households living in crowded housing units	85.5	0.000245* (0.0000345)	–0.0139* (0.00378)
Non-employed for population 16–64 years	441	0.000586* (0.0000343)	0.00620* (0.00156)

The model controlled for the year, prescriber gender, specialty, provider experience, graduate school rank, teaching location, metropolitan area, US state, and beneficiary characteristics (demographics, risk scores, and dual public insurance).

* indicates a *P* value < 0.0005

# Log-transformed values (using the natural logarithm) were used to normalize the data.

^ Used the normalized scores for SDI components rather than percentages

$ The Weak instrument *F* value >10 suggests strong instruments.

## Discussion

To our knowledge, this is the first study to investigate a potential causal relationship between socioeconomic deprivation and antibiotic prescription. Higher SDI was associated with increased antibiotic days supplied, claims, and cost per 100 beneficiaries, but no change in days per claim. Using the maximum EITC as a valid instrument for predicting SDI, the IV approach addressed endogeneity and uncovered causality.^24^

Our findings contrast with other studies that assessed non-causal associations between SDI and antibiotic use. For example, Kissler *et al.* ^8^ found fewer upper respiratory infection visits among Massachusetts residents with high SDI, while our previous study in Texas showed lower fluoroquinolone use with higher SDI.^5^

Other indices, such as the neighborhood deprivation index,^26^ area deprivation index,^27^ and social vulnerability index,^28^ have been used to evaluate deprivation and healthcare outcomes. We chose SDI for its simplicity, availability, and ability to aggregate data at the census tract level, which supported robust analysis. The maximum EITC further helped address potential endogeneity.

Spatial analyses revealed geographic clustering of SDI and antibiotic days supplied, but the bivariate spatial association was non-significant. High-high SDI clusters were concentrated in the South, while high-high antibiotic use clusters were more common in the Midwest, resulting in a non-significant bivariate spatial association. These findings suggest that regional factors, such as healthcare access or patient behavior, may contribute to differences in the SDI-antibiotic use relationship. The IV model addressed this heterogeneity and isolated the causal relationship.

As an exploratory analysis, we examined the unadjusted association between EITC and antibiotic use, which was statistically significant (estimate –0.0379, *P* < 0.00005). However, this association does not account for unmeasured confounders, such as healthcare access, provider preferences, or patient comorbidities. This highlights the potential for bias in standard regression models and underscores the importance of using an IV approach to better isolate the relationship between SDI and antibiotic prescribing.

The effect of SDI on antibiotic use was multifaceted. Census tracts with more single-parent families and unemployed adults had higher antibiotic use, while crowded housing was associated with lower use, possibly due to limited healthcare access. Practices serving larger percentages of Black and Hispanic beneficiaries prescribed fewer antibiotics and incurred lower costs, potentially reflecting healthcare access barriers or provider-patient interaction challenges. However, race and ethnicity were confounders in the IV model, preventing causal conclusions.

This study has several limitations. The CMS repositories lacked information on antibiotic indications, making it impossible to assess prescription appropriateness or health outcomes. High prescription rates might reflect overuse, while low rates could indicate barriers to healthcare access, but these patterns cannot be fully quantified. Although we adjusted for area-, provider-, and patient-level confounders, key variables such as healthcare access, comorbidities, and patient education were not included, potentially reducing accuracy.

The 10-year study period included significant socioeconomic changes, especially during the COVID-19 pandemic, disproportionately affecting lower socioeconomic groups and likely influencing antibiotic prescribing and SDI metrics. This temporal variability, combined with the lack of year-by-year sub-analyses, limits our ability to determine whether patterns observed are driven by specific periods, such as the pandemic, or represent broader trends. Additionally, SDI was tied to the provider’s census tract rather than the patient’s, and imputation was used for some years based on the nearest available data. While practical, this approach was not extensively validated, and missing data frequency was not quantified, possibly introducing bias. The assumption that patients reside near their provider may not always hold, and the correlation between provider and patient SDI remains unassessed.

Despite these limitations, the study has key strengths. Its national scope over a decade makes findings generalizable to outpatient practitioners caring for older patients. By analyzing multiple prescription metrics (e.g., days supplied, claims, and cost), we captured trends and variations comprehensively. While a standard single-stage model would examine the direct association between SDI and antibiotic use, our IV approach—leveraging EITC as an exogenous proxy—allows us to address potential endogeneity concerns. This method, combined with adjustment for key confounders, enhances the validity of our findings. Additionally, a stringent P-value cut-off (0.0005) minimized type I error.

Our study provides critical public health insights. It highlights census tracts with high antibiotic utilization, enabling targeted interventions. Additionally, it identifies SDI categories influencing prescribing patterns, offering opportunities to address knowledge gaps among patients and providers in areas with high single-parent households, unemployment, or barriers to access, such as crowded housing. These findings underscore the role of healthcare providers and systems in reducing antibiotic misuse and promoting equitable access to care in high-SDI areas.

In conclusion, our study suggests that socioeconomic deprivation may influence antibiotic prescribing rate. Certain components of SDI may drive antibiotic over- or under-utilization. This demonstrates the complexity of the relationship and the need for further investigation into these mechanisms to target public health interventions. Moreover, it indicates that a one-size-fits-all approach is unlikely to address the problem of antibiotic misuse effectively.

## Supporting information

Al Mohajer et al. supplementary material Al Mohajer et al. supplementary material

## Supplementary material

To view supplementary material for this article, please visit https://doi.org/10.1017/ash.2025.185
