# Supplementary figures and images for: Investigating socioeconomic deprivation and antibiotic prescribing among older medicare patients using an instrumental variable approach
Source: Antimicrob Steward Healthc Epidemiol. 2025 May 13;5(1):e110. doi: 10.1017/ash.2025.185 (PMC12089662; doi:10.1017/ash.2025.185)

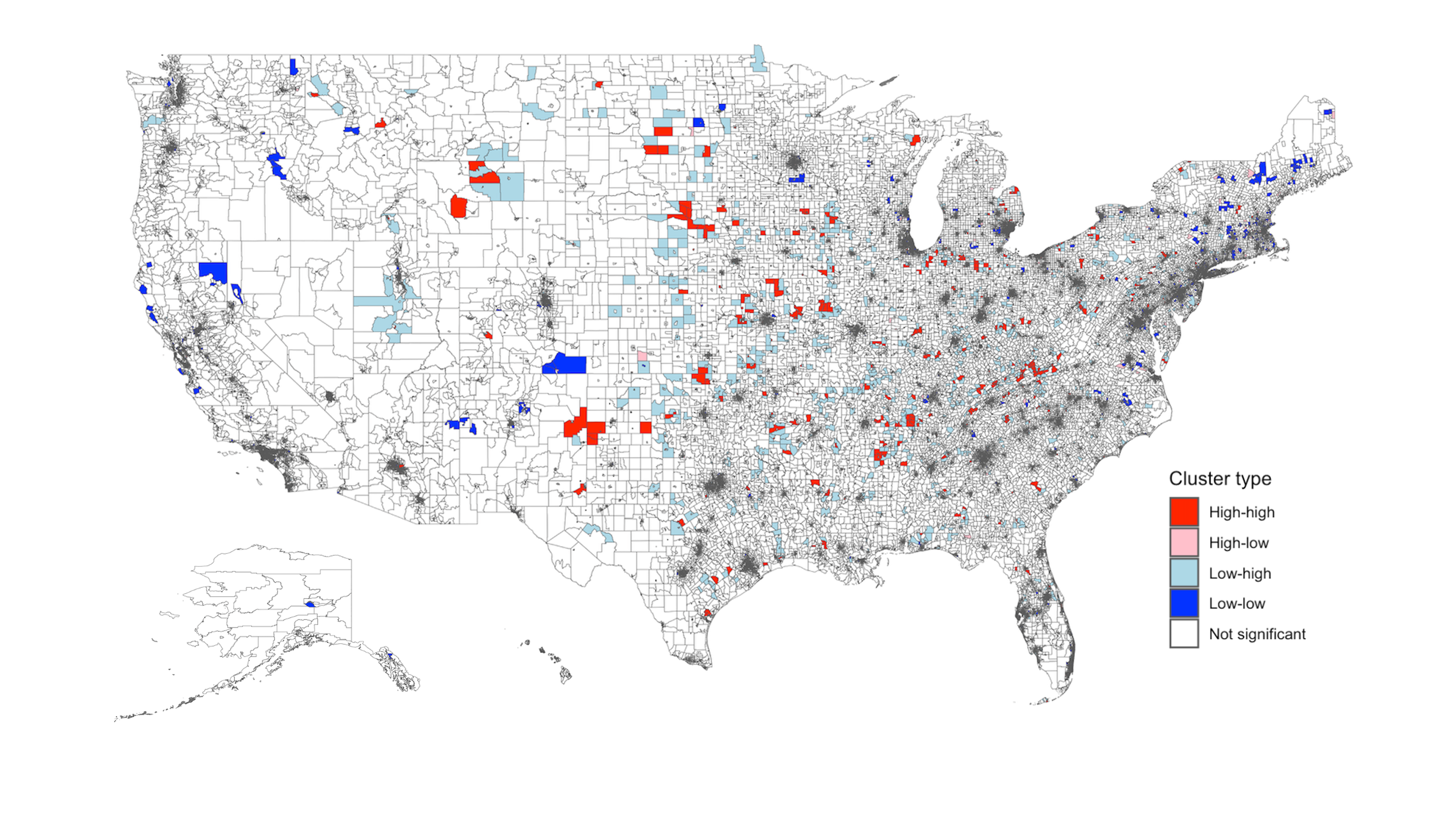

Supplement: Al Mohajer et al. supplementary material [file S2732494X25001858sup001.tiff]
